# Atrial Septal Defect with Eisenmenger Syndrome: A Rare Presentation

**DOI:** 10.1155/2020/8681761

**Published:** 2020-03-09

**Authors:** Arnold Nongmoh Forlemu, Muhammad Ajmal, Mehrdad Saririan

**Affiliations:** ^1^Creighton University School of Medicine-Phoenix/Maricopa Integrated Health System, Department of Internal Medicine-Cardiology, 2601 E Roosevelt St., Phoenix, AZ 85008, USA; ^2^University of Arizona College of Medicine-Tucson, Sarver Heart Center, Department of Cardiology, 750 W Orange Grove Road, Tucson, AZ 85704, USA; ^3^Creighton University School of Medicine-Phoenix/Maricopa Integrated Health System, Department of Cardiology, 2601 E Roosevelt St., Phoenix, AZ 85008, USA

## Abstract

Atrial septal defects (ASDs) are common congenital heart defects (CHD). The clinical course in patients without closure of the ASD is associated with significant morbidity and mortality in advanced age. A small percentage of patients may develop pulmonary arterial hypertension (PAH) due to left to right shunting that impacts morbidity and mortality. Advances in prenatal screening and fetal echocardiography have allowed timely interventions. Nonetheless, some patients still may be diagnosed with ASD in adulthood as an incidental finding or presenting with clinical symptoms such as shortness of breath from right heart failure. We report a case of an adult female presenting with shortness of breath due to ASD causing PAH with Eisenmenger physiology.

## 1. Introduction

Atrial septal defects (ASDs) are the second most common congenital heart defects (CHD) [1]. It accounts for about 13% of CHD cases with a 2 : 1 female to male ratio. The most common type of ASD is the secundum type that is located at the fossa ovalis [2]. ASDs may be benign, but occasionally left to right shunting can overload the right heart leading to right heart failure (RHF) and arrhythmias. In its advanced form, Eisenmenger syndrome (ES) with shunt reversal may develop, with a worse prognosis [2]. Shunt closure is indicated in the presence of severe shunting with signs of RHF with a pulmonary vascular resistance < 5 Wood units [3]. However, when irreversible pulmonary arterial hypertension (PAH) and ES develop, closure is contraindicated and medical management becomes the focus [3].

## 2. Case Report

**Figure 1:**
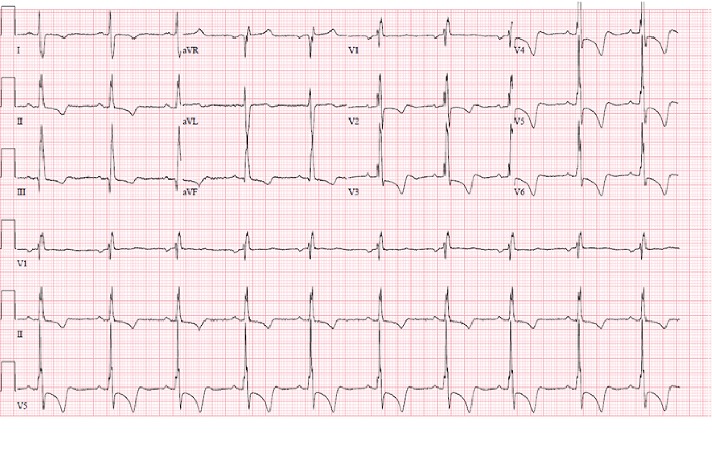
Electrocardiogram demonstrating Right Ventricular Hypertrophy Pattern.

A 44-year-old female with a history of heart murmur since age 15 and chronic dyspnea on exertion was admitted to the hospital with new-onset chest pain and shortness of breath. The pain was retrosternal, worse when lying flat, and improved with leaning forward. She was hypoxic on admission with an oxygen saturation of 86%. Physical examination revealed cyanosis, jugular venous distention, a right ventricular heave, and a loud pulmonic diastolic murmur. Blood work revealed a hemoglobin concentration of 14.2 g/dl, a normal troponin level < 0.012 ng/ml, serum creatinine of 0.51 mg/dl, negative antinuclear antibodies, negative HIV serology, and a negative pregnancy test. An electrocardiogram showed severe right ventricular hypertrophy (Figure 1).

**Figure 2:**
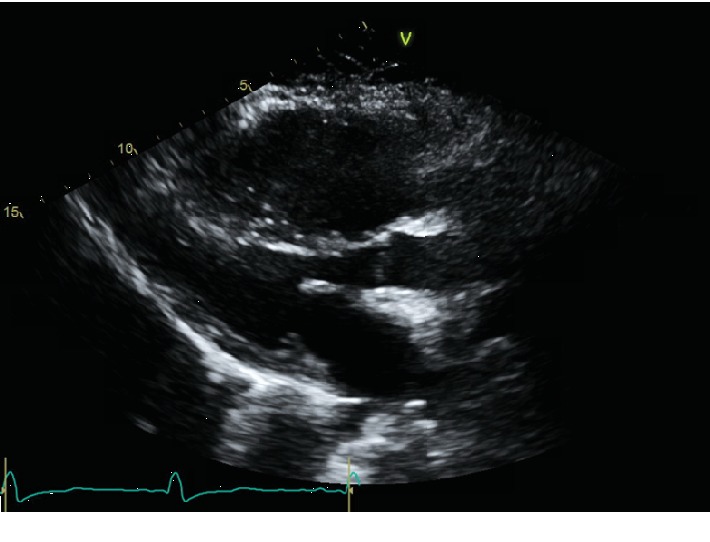
Transthoracic Echocardiogram Parasternal Long Axis View showing enlarged right ventricle from volume overload.

Echocardiography revealed severe right atrial and right ventricular enlargement, severe pulmonary hypertension with right ventricular systolic pressure (RSVP) of 70 mmHg, and suspicion for a large ASD (Figure 2).

She underwent right heart catheterization (RHC) with a shunt run. This revealed 10% oxygen step-up from high superior vena cava to the right atrium, suggestive of ASD. Effective pulmonary blood flow to systemic blood flow (Qp/Qs) was 1, suggestive of equal bidirectional shunting and Eisenmenger physiology. RHC confirmed severe PAH (PVR = 8.7 Wood units) that did not respond to inhaled nitric oxide. The mean wedge pressure was 2 mmHg. She also underwent a computed tomography (CT) pulmonary angiogram that was negative for thromboembolic disease. A pulmonary function test (PFT) and diffusion lung capacity for carbon monoxide (DLCO) were normal.

**Figure 3:**
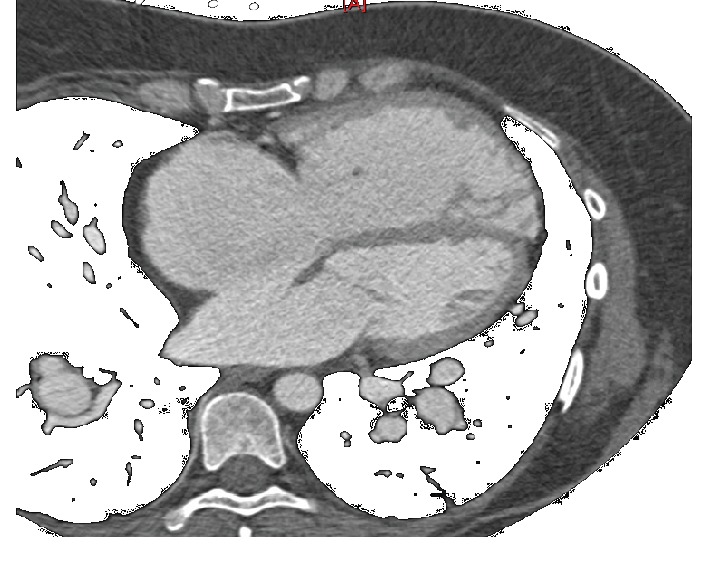
Cardiac CT showing large ASD.

To better delineate the anatomy of the atrial septum, she underwent cardiac CT which revealed a large (2.5 × 3.5 cm) secundum-type defect (Figure 3).

She had a six-minute walk test for prognostic purposes which revealed reduced walk distance of 300 meters or 50% of the predicted distance. She was classified as WHO class III functional status and started on combination therapy with Sildenafil 20 mg three times daily and Macitentan 10 mg once daily. She was also placed on two forms of contraception (barrier and nonestrogen contraceptives). Her shortness of breath and chest pain improved significantly, and she was scheduled for outpatient follow-up with a pulmonologist and cardiologist. At 30 days, the patient no longer felt short of breath with activities of daily living and her 6-minute walk test doubled to 600 meters.

## 3. Discussion

ASD is often encountered in the adult population, as many patients are symptom-free in the first few decades of life. Secundum-type ASDs are located at the fossa ovalis and represent about 70% of all ASDs [2]. To determine shunt direction during RHC, the superior vena cava sample (SVC) for venous oxygen saturation is best taken at high-level SVC to avoid contamination with mixed venous oxygen in the lower level SVC blood, owing to the latter being closer to the right atrium and the inferior vena cava that may underestimate left-right shunting.

PAH is characterized by a pulmonary capillary wedge pressure ≤ 15 mmHg and a pulmonary vascular resistance (PVR) > 3 Wood units (WU). Cardiac output and PVR are often measured by thermodilution. However, this technique may be inaccurate in patients with intracardiac shunts, low cardiac output states, or significant tricuspid regurgitation. In such situations, the Fick principle may be preferable to calculate cardiac output and thus PVR [4].

PAH in secundum-type ASD seems to be related to age, size of the defect, female sex, and nonclosed ASD status [5, 6]. ASD recognition and correction early after childbirth has helped reduce the incidence of PAH [5]. Unrecognized ASD often presents in the 4^th^ decade of life (as it did with our patient) with dyspnea on exertion, chest pain, palpitations, and sometimes RHF [6]. Severe pulmonary vascular disease with ES is very rare (<5%) and may be seen in those with genetic predispositions, like in idiopathic PAH [6].

Whether ASD ES is secondary to severe PAH due to the ASD shunt size alone or the ES is related to idiopathic PAH with a standby ASD is still a subject of debate. Therrien et al. [7] demonstrated the absence of bone morphogenetic protein receptor 2 (BMPR2) gene mutation in patients with ASD ES, suggesting ASD size plays a major role in ES development. The BMPR2 gene mutation is found in 70% of patients with familial PAH and 25% of patients with idiopathic PAH. The BMPR2 gene mutation has been implicated in abnormal vascular smooth muscle cell proliferation and endothelial apoptosis [7]. Patients presenting in their teenage years with PAH and ASD with BMPR2 mutation may have abnormal cell signaling from the mutation coupled with hemodynamic overload from the ASD promoting rapid progression of pulmonary vascular lesions resulting in severe PAH [7]. Although the BMPR2 gene mutation was absent in the above study, patients with ASD may still carry an unknown genetic mutation predisposing them to PAH ES, which may become clinically apparent when triggered by an external stimulus such as high flow through large shunt size. This may explain why ASD patients rarely develop ES, as the genetic predisposition, if any, likely occurs rarely in the population or has a low penetrance in society.

In the study by Therrien et al. [7], patients with ASD ES also had significantly larger ASDs than the control patients (3.7 ± 1.2 cm versus 1.9 ± 0.7 cm). The patient in our case presented in her 4^th^ decade with PAH ES and a large ASD (2.5 × 3.5 cm) suggesting that the magnitude of the ASD likely played a major hemodynamic role leading to shunt reversal and ES.

It is worth noting that other conditions can cause precapillary PH, with the most common being left heart diseases such as diastolic heart failure [8]. Also, precapillary PH in the presence of an ASD can be caused by obstructive lung disease, pulmonary fibrosis, and thromboembolism [8]. The patient in our case had a normal wedge pressure, normal PFT and DLCO, and a step-up of oxygen saturation from high superior vena cavae to the right heart chambers with bidirectional ASD shunting, all pointing towards an ASD as being the primary cause of the PAH.

## 4. Management

Secundum ASDs can be closed either surgically or percutaneously, and repair is indicated when there is evidence of RV compromise with or without symptoms [6]. Left to right shunt with Qp : Qs ≥ 1.5 and PVR < 2.3 Wood units (or PVRi < 4 WU/m^2^) is safe for ASD closure. Closure of the defect and pregnancy are not recommended if the PVR is >4.6 WU (PVRi > 8 WU/m^2^), even when an Eisenmenger-type phenomenon has not been reached. For patients with PVR between 2.3 and 4.6 WU, the decision to close the ASD is made on a case-by-case basis. Once the shunt has reversed from right to left (Qp : Qs < 1) or is bidirectional (Qp : Qs = 1), PAH is often irreversible and ASD closure is contraindicated due to worsening RHF [6, 8]. This is because closing the shunt in such instances may sacrifice the right-sided pressure relief role that the ASD may be providing. Preserving right ventricular function by upfront combination therapy or by escalating pulmonary vasodilators can significantly improve patient outcomes when ES develops. As such, phosphodiesterase-type 5 inhibitors, endothelin receptor antagonists, prostacyclin analogues, and receptor agonists, either with upfront combination therapy or with sequential escalation of monotherapy, are recommended [9, 10]. The “treat and repair” strategy in which PVR is lowered enough by medications to allow for shunt closure remains controversial. Some authors have reported successful shunt closure without complications in patients with significant PVR after lowering the PVR to <4-6.5 WU with medical management [9, 11]. However, this approach is not currently supported by guidelines and could be potentially fatal.

Pregnancy is contraindicated in all forms of PAH due to risk of cardiac, fetal, and obstetric complications [10, 12]. Women of childbearing age found to have PAH should be placed on two different forms of contraceptive (excluding estrogen-containing products due to increased thromboembolism risk) before starting medical management, including a barrier method and nonestrogen contraceptives [10, 12].

## 5. Conclusion

ASDs are not commonly associated with Eisenmenger physiology. The morbidity and mortality in ASD-related PAH are due to cardiac complications including heart failure and arrhythmias. Once ES develops, ASD closure is contraindicated. Medical management and avoidance of situations that can alter hemodynamic balance (such as pregnancy) to reduce right-sided pressures are paramount to the patient's survival.

## Additional Points

*Learning Objective*. ASDs are not commonly associated with Eisenmenger physiology, mostly owing to volume rather than pressure loading on the pulmonary circulation as well as early recognition and closure of ASD. Once ES develops, timely medical management with vasodilators, postponing closure, and avoidance of situations that alter hemodynamic balance (e.g., pregnancy) become paramount to the patient's survival.

## Supplementary Materials

Supplementary Materials “Detailed data on blood work, echocardiography, and right heart catheterization.”

## References

[1] van der Linde D., Konings E. E., Slager M. A. (2011). Birth prevalence of congenital heart disease worldwide: a systematic review and meta-analysis. *Journal of the American College of Cardiology*.

[2] Goetschmann S., Dibernardo S., Steinmann H., Pavlovic M., Sekarski N., Pfammatter J. P. (2008). Frequency of Severe Pulmonary Hypertension Complicating "Isolated" Atrial Septal Defect in Infancy. *The American Journal of Cardiology*.

[3] van de Veerdonk M. C., Kind T., Marcus J. T. (2011). Progressive right ventricular dysfunction in patients with pulmonary arterial hypertension responding to therapy. *Journal of the American College of Cardiology*.

[4] Nishimura R. A., Carabello B. A. (2012). Hemodynamics in the cardiac catheterization laboratory of the 21^st^ century. *Circulation*.

[5] Vogel M., Berger F., Kramer A., Alexi-Meshkishvili V., Lange P. E. (1999). Incidence of secondary pulmonary hypertension in adults with atrial septal or sinus venosus defects. *Heart*.

[6] Baumgartner H., Bonhoeffer P., de Groot N. M. (2010). ESC guidelines for the management of grown-up congenital heart disease (new version 2010). *European Heart Journal*.

[7] Therrien J., Rambihar S., Newman B. (2006). Le syndrome d'Eisenmenger et les communications interauriculaires : Inne ou acquis?. *Canadian Journal of Cardiology*.

[8] Nashat H., Montanaro C., Li W. (2018). Atrial septal defects and pulmonary arterial hypertension. *Journal of Thoracic Disease*.

[9] Kijima Y., Akagi T., Takaya Y. (2016). Treat and repair strategy in patients with atrial septal defect and significant pulmonary arterial hypertension. *Circulation Journal*.

[10] Galiè N., Humbert M., Vachiery J. L. (2015). 2015 ESC/ERS guidelines for the diagnosis and treatment of pulmonary hypertension: the joint task force for the diagnosis and treatment of pulmonary hypertension of the European Society of Cardiology (ESC) and the European Respiratory Society (ERS) endorsed by: Association for European Paediatric and Congenital Cardiology (AEPC), International Society for Heart and Lung Transplantation (ISHLT). *European Respiratory Journal*.

[11] Bradley E. A., Ammash N., Martinez S. C. (2019). "Treat-to-close": non-repairable ASD-PAH in the adult: results from the North American ASD-PAH (NAAP) multicenter registry. *International Journal of Cardiology*.

[12] Ladouceur M., Benoit L., Radojevic J. (2017). Pregnancy outcomes in patients with pulmonary arterial hypertension associated with congenital heart disease. *Heart*.

