# Supplementary material for: Atrial Septal Defect with Eisenmenger Syndrome: A Rare Presentation
Source: Case Rep Cardiol. 2020 Mar 9;2020:8681761. doi: 10.1155/2020/8681761 (PMC7085360; doi:10.1155/2020/8681761)
Supplement: Supplementary Materials — “Detailed data on blood work, echocardiography, and right heart catheterization.” [file 8681761.f1.pdf]

Right heart catheterization data

Right Atrium: mean 0 mmHg
Right ventricle: 84/2 mmHg
Pulmonary artery: 83/27, mean 47 mmHg
Pulmonary capillary wedge: 2/4, mean 2 mmHg
Cardiac output: 5.2 L/min
Cardiac index: 3.2 L/min/m2
Pulmonary vascular resistance: 8.7 Wood units

No response to Inhaled Nitric Oxide up to 80 per million:
Pulmonary artery (20 ppm): 76/23, mean 43 mmHg
Pulmonary artery (40 ppm): 75/22, mean 43 mmHg
Pulmonary artery (80 ppm): 75/23, mean 43 mmHg

Pulmonary artery: 71%
Mid right atrium: 70%
Superior vena cava: 60%
Inferior vena cava 59%
Pulmonary venous (assumed): 99%
Systemic (aorta): 89%
pulmonary blood flow to systemic blood flow ratio Qp/Qs= 1.0

Blood data

White blood cell count	$9.2x10^3$/ul
Hemoglobin concentration	14.2g/dl
Platelet count	208k/ul
International Normalized ratio, INR	1.1
Sodium	139 mmol/L
Potassium	4.0 mmol/L
Blood Urea Nitrogen	18 mg/dL
Creatinine	0.51 mg/dL
Glucose	104 mg/dL
B-type Natriuretic Peptide	702 pg/mL
Troponin	<0.012 ng/mL
Troponin 4 hours later	<0.012 ng/mL
Tyrosine stimulating hormone	1.098 uIU/mL
Alanine aminotransferase	30U/l
Aspartate aminotransferase	46U/l
Total bilirubin	0.6mg/dl
Hepatitis C antibody	Negative
HIV antigen/antibody screen	Negative
Beta HCG qualitative	Negative
Antinuclear antibody, ANA	Negative
C reactive protein, high sensitivity	1.6mg/L

Erythrocyte sedimentation rate	26mm/hour
D dimer	<230 D-DU ng/mL

Transthoracic echocardiographic data

IVSd: 0.72 cm
LVIDd: 4.0 cm
EDV(Teich): 70.40 ml
LVPWd: 0.72 cm
LVd Mass (ASE): 81.89 g
LVd Mass Ind (ASE): 50.24 g/m^2
RWT: 0.36
LVIDs: 3.0 cm
ESV(Teich): 34.74 ml
EF(Teich): 50.65 %
ESV(Cube): 26.76 ml
EF(Cube): 58.49 %
%FS: 25.40 %
SV(Teich): 35.65 ml
SI(Teich): 21.87 ml/m^2
SV(Cube): 37.70 ml
SI(Cube): 23.13 ml/m^2
LVOT Diam: 1.83 cm
LA Diam: 4.34 cm
Ao asc: 3.09 cm
Ao st junct: 2.61 cm
RVIDd: 4.70 cm
Ao sinus: 2.48 cm
RVA (d): 51.91 cm^2
RVA (s): 37.17 cm^2
RV FAC: 2839 %
RA Area: 31.19 cm^2

LALs A4C: 6.16 cm
LAAs A4C: 18.98 cm^2
TAPSE: 1.6 cm
RVOT maxPG: 0.06 mmHg
RVOT Vmax: 0.13 m/s
MV E Vel: 0.61 m/s
MV DecT: 192.37 ms
MV Dec Slope: 3.19 m/s^2
MV A Vel: 0.69 m/s
MV E/A Ratio: 0.89
LVOT Vmax: 1.0 m/s
LVOT Vmean: 0.63 m/s
LVOT maxPG: 3.78 mmHg
LVOT meanPG: 1.87 mmHg
LVOT Env.Ti: 299.71 ms
LVOT VTI: 18.8 cm
HR: 61 BPM
LVSV Dopp: 49.65 ml
LVSI Dopp: 30.46 ml/m^2
LVCO Dopp: 3.02 l/min
LVCI Dopp: 1.85 $l/minm^2$
AV Vmax: 1.3 m/s
AV Vmean: 1.0 m/s
AV maxPG: 7 mmHg
AV meanPG: 4 mmHg
AV Env.Ti: 296.9 ms
AV VTI: 28.8 cm

HR: 60 BPM
AVA Vmax: 1.91 cm^2
AVA (VTI): 1.72 cm^2
AVAI (VTI): 1.058 cm^2/m^2
AVAI Vmax: 1.174 cm^2/m^2
P Vein S: 0.51 m/s
P Vein D: 0.33 m/s
P Vein S/D Ratio: 1.55
P Vein A: 0.25 m/s
P Vein A Dur: 108.47 ms
RVOT Vmax: 0.65 m/s
RVOT maxPG: 1.67 mmHg
PV Vmax: 1.42 m/s
PV maxPG: 8.11 mmHg
PAEDP: 22.99 mmHg
PRend Vmax: 2.23 m/s
PRend PG: 19.99 mmHg
TR Vmax: 4.2 m/s
TR maxPG: 70 mmHg
RAP: 3 mmHg
RVSP: 73 mmHg
AV DI: 0.65
